# Management of respiratory distress following prehospital implementation of noninvasive ventilation in a physician-staffed emergency medical service: a single-center retrospective study

**DOI:** 10.1186/s13049-021-00900-7

**Published:** 2021-06-29

**Authors:** Adeline Dunand, Nicolas Beysard, Ludovic Maudet, Pierre-Nicolas Carron, Fabrice Dami, Lise Piquilloud, David Caillet-Bois, Mathieu Pasquier

**Affiliations:** 1 Faculty of Biology and Medicine, University of Lausanne, Rue du Bugnon 21, 1011 Lausanne, Switzerland; 2 Department of Emergency Medicine, Lausanne University Hospital, Rue du Bugnon 46, 1011 Lausanne, Switzerland; 3 Adult Intensive Care Unit, Lausanne University Hospital, Rue du Bugnon 46, 1011 Lausanne, Switzerland

**Keywords:** Acute pulmonary edema, Acute respiratory failure, Chronic obstructive pulmonary disease exacerbation, Emergency medical services, Noninvasive ventilation, Prehospital, Respiratory distress

## Abstract

**Background:**

Noninvasive ventilation (NIV) is recognized as first line ventilatory support for the management of acute pulmonary edema (APE) and chronic obstructive pulmonary disease (COPD) exacerbations. We aimed to study the prehospital management of patients in acute respiratory distress with an indication for NIV and whether they received it or not.

**Methods:**

This retrospective study included patients ≥18 years old who were cared for acute respiratory distress in a prehospital setting. Indications for NIV were oxygen saturation (SpO_2_) <90% and/or respiratory rate (RR) >25/min with a presumptive diagnosis of APE or COPD exacerbation. Study population characteristics, initial and at hospital vital signs, presumptive and definitive diagnosis were analyzed. For patients who received NIV, dyspnea level was evaluated with a dyspnea verbal ordinal scale (D-VOS, 0-10) and arterial blood gas (ABG) values were obtained at hospital arrival.

**Results:**

Among the 187 consecutive patients included in the study, most (*n* = 105, 56%) had experienced APE or COPD exacerbation, and 56 (30%) received NIV. In comparison with patients without NIV, those treated with NIV had a higher initial RR (35 ± 8/min vs 29 ± 10/min, *p* < 0.0001) and a lower SpO_2_ (79 ± 10 vs 88 ± 11, *p* < 0.0001). The level of dyspnea was significantly reduced for patients treated with NIV (on-scene D-VOS 8.4 ± 1.7 vs 4.4 ± 1.8 at admission, *p* < 0.0001). Among the 131 patients not treated with NIV, 41 (31%) had an indication. In the latter group, initial SpO_2_ was 80 ± 10% in the NIV group versus 86 ± 11% in the non-NIV group (*p* = 0.0006). NIV was interrupted in 9 (16%) patients due to either discomfort (*n* = 5), technical problem (*n* = 2), persistent desaturation (*n* = 1), or vomiting (*n* = 1).

**Conclusions:**

The results of this study contribute to a better understanding of the prehospital management of patients who present with acute respiratory distress and an indication for NIV. NIV was started on clinically more severe patients, even if predefined criteria to start NIV were present. NIV allows to improve vital signs and D-VOS in those patients. A prospective study could further elucidate why patients with a suspected diagnosis of APE and COPD are not treated with NIV, as well as the clinical impact of the different strategies.

**Trial registration:**

The study was approved by our institutional ethical committee (CER-VD 2020-01363).

## Background

Acute respiratory distress is one of the most frequent conditions encountered by physician-staffed emergency medical services (PEMS), often due to acute pulmonary edema (APE) or chronic obstructive pulmonary disease (COPD) exacerbation [1–3]. Current recommendations for in-hospital treatment of APE and COPD exacerbation support the use of noninvasive ventilation (NIV) as first-line supportive treatment in addition to standard care based on oxygen and medication [4–6]. NIV has been shown to be feasible in the prehospital setting, to improve the clinical status of the patient at hospital arrival, and to reduce intubation rate and intensive care unit (ICU) admission [7–11].

On January 1, 2019, a compact turbine double limb circuit ventilator (Monnal T60, Air Liquide Medical Systems SA, Antony, France) was implemented in the Lausanne University Hospital PEMS and criteria to initiate NIV in the prehospital setting were defined. To our knowledge, no study has compared patients treated with NIV to those who are eligible for this therapy without receiving it in the prehospital setting. We aimed to study the management of patients with respiratory distress in the context of the implementation of a prehospital NIV protocol and whether they received this treatment or not.

## Method

### Setting

The PEMS of Lausanne University Hospital serves an urban (150 km^2^) and suburban (400 km^2^) area with a population of about 295,000 inhabitants [12]. The dispatch center uses a criteria-based system to accommodate the deployment of rescue. EMS are staffed with at least one paramedic and either another paramedic or an emergency medical technician. The paramedics’ “dyspnea” algorithm allows them to administer oxygen and inhaled salbutamol. The emergency physician may be dispatched by ground to the site to strengthen the prehospital care either directly by the dispatch center or at the request of a paramedic on site. Prehospital emergency physicians have advanced airways management capabilities and can administer different drugs, notably isosorbide dinitrate, furosemide, methylprednisolone, morphine, and salbutamol. The double limb circuit compact transport turbine ventilator (Monnal T60, Air Liquide Medical Systems SA, Antony, France) allows both invasive ventilation and NIV, providing either of 2 modes: continuous positive airway pressure or bi-level positive airway pressure (BiPAP) [13]. A large adult face mask size 6 (Vigon, Ecouen, France) is used. BiPAP was the mode of NIV used at the initiation of treatment. The criteria for prehospital NIV use in our PEMS for patients with a presumed diagnosis of APE or COPD exacerbation are an oxygen saturation (SpO_2_) <90% and/or a respiratory rate (RR) >25/min and/or the use of accessory muscles [4, 8, 14]. We do not perform ABGs in the prehospital setting and recommend the use of BiPAP for suspected APE as well as COPD exacerbation [4]. The physicians are either advanced trainees (about 50%) or board-certified (about 50%) physicians in emergency medicine (about 50%) or anesthesiology (about 50%). Each physician followed a dedicated 1-hour teaching session, including the Monnal T60 use and the criteria for the prehospital use of NIV. For every patient, an electronic prehospital chart (Attrib, iMatt Sàrl, Boudevilliers, Switzerland) is filled out by the prehospital physician at the end of the rescue mission which contains prehospital contextual and clinical information, as well as the 48-hour hospital diagnosis and outcome. In addition, specific information is prospectively collected for each patient for whom NIV has been considered and stored on RedCap (Vanderbilt University, Nashville, TN, USA).

### Study design

In this retrospective monocentric study, we included consecutive patients ≥18 years old who were managed by our PEMS from January 1, 2019, to December 31, 2019 with at least one of the following characteristics: a reported dyspnea or signs of acute respiratory distress, a presumptive or definitive diagnosis of APE or COPD exacerbation, or those for whom NIV was considered. According to our ethical committee’s requirements, all patients with inclusion criteria were considered except those who didn’t sign the institutional general research consent. The study was approved by our institutional ethical committee (CER-VD 2020-01363).

We report the following characteristics of the included patients : age and gender; prehospital time intervals; clinical parameters on arrival of the PEMS on scene and at hospital admission (RR, SpO_2_, heart rate (HR), systolic and diastolic blood pressures (SBP and DBP), temperature, Glasgow Coma Scale (GCS) ; prehospital presumed diagnosis; prehospital treatments including drugs provided, bag-mask ventilation or intubation; whether the patient was admitted to an intermediate care unit or an ICU; and 48-hour hospital mortality and diagnosis. The severity of involvement was graded by the prehospital emergency physician according to the 8-level National Advisory Committee for Aeronautics (NACA) score [15].

Specific data recorded for patients for whom NIV had been considered were as follows: indications and contraindications to NIV, main settings of the NIV (positive end expiratory pressure (PEEP), pressure support (PS), and fraction of inspired oxygen (FiO_2_)) at the beginning of NIV and at hospital admission, and whether NIV had to be stopped before hospital admission and the reason why. Dyspnea was graded in patients who received NIV by using the 11-level dyspnea verbal ordinal scale (D-VOS) on site and at hospital admission [16].

We also report the first arterial blood gas (ABG) performed at hospital admission and whether the patient was admitted to the ICU within the first hour after admission for that subpopulation.

Our primary outcome was the comparison of the patients meeting criteria for NIV, according to whether they received prehospital NIV or not. Secondary outcomes included the NIV failure rate, defined as premature interruption of treatment, and its causes as well as the evolution of the D-VOS score in the NIV group.

### Statistical analysis

The collected data were exported from RedCap to Stata version 14 (Stata Corporation, College Station, TX, USA). Continuous variables were expressed as mean and standard deviation (SD) when the data were normally distributed or as median and interquartile range (IQR) when non-normally distributed. Categorical variables were reported as absolute number and relative percentage. Student's *t*-test was used to compare continuous and normally distributed data, and Wilcoxon-Mann-Whitney test was used for continuous and non-normally distributed data. Pearson’s chi-square test and Fisher's exact test were used for categorical variables as requested. A two-tailed *p*-value of <0.05 was considered as statistically significant.

## Results

**Fig. 1:**
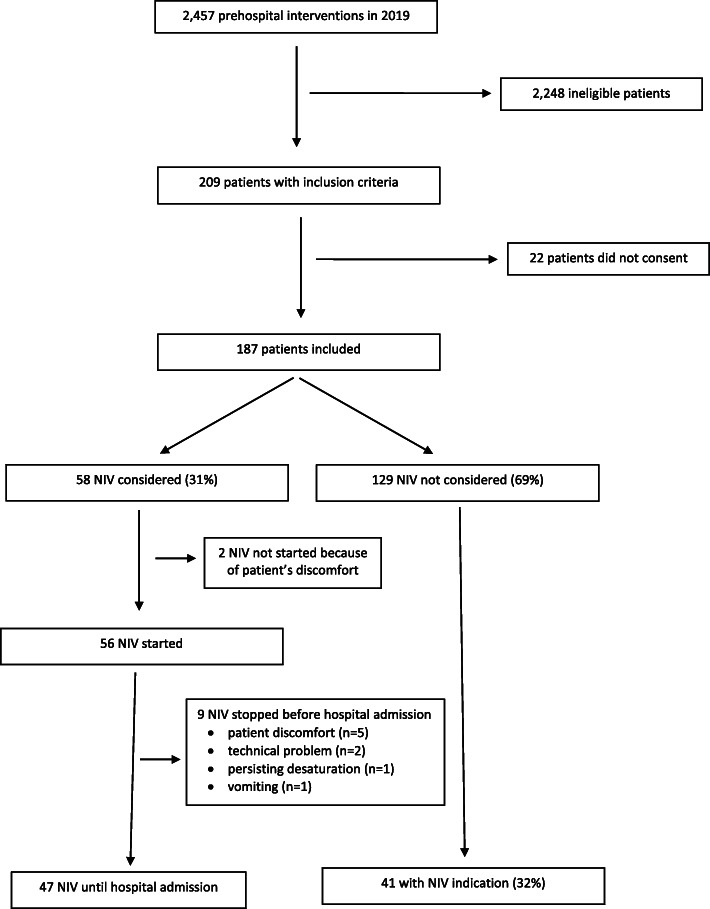
Flowchart of study patients

Among the 2,457 patients rescued by our PEMS in 2019, 187 met the inclusion criteria (Fig. 1). Of these, NIV was anticipated in 58 (31%) patients and actually started in 56 (30%). Indeed 2 patients had a contraindication to NIV because of noncompliance and discomfort. Frequency of NIV use was constant throughout the months of the year (*p* = 0.35). Table 1 shows the general and clinical characteristics of the patients in the NIV and non-NIV groups.

**Table 1:** Characteristics of the overall study population (*n* = 187)

		Total (*n* = 187)	NIV started (*n* = 56; 30%)	NIV not started (*n* = 131; 70%)	***p*** Value
**Characteristic**					
Age (year), mean ± SD		77 ± 14	77 ± 12	76 ± 15	0.96
Male, n (%)		96 (51)	28 (50)	68 (52)	0.8
NACA, n (%)					<0.0005
	2	2 (1.1)	0 (0)	2 (1.5)	
	3	40 (21)	0 (0)	40 (31)	
	4	69 (37)	17 (30)	52 (40)	
	5	71 (38)	38 (68)	33 (25)	
	6	4 (2.1)	1 (1.8)	3 (2.3)	
	7	1 (0.5)	0 (0)	1 (0.8)	
**Time interval (minutes), median (IQR)**					
Time to start		2.7 (1.9–3.7)	2.9 (2.0–4.0)	2.7 (1.8–3.6)	0.32
Time to scene		5.9 (4.1–8.1)	5.3 (3.5–7.2)	6.0 (4.4–8.2)	0.07
On-scene time		21 (16–26)	22 (18–27)	20 (15–25)	0.08
Transport		10.4 (3.1–16.8)	7.6 (4.4–11.0)	12.7 (7.1–20.1)	<0.0001
**Presumptive diagnosis, n (%)**					
	APE^a^	57 (30)	30 (55)	27 (21)	<0.0001
	COPD^a^ exacerbation	48 (26)	20 (36)	28 (21)	0.04
	Pneumonia	53 (28)	15 (27)	38 (29)	0.76
	ARDS	1 (0.5)	1 (1.8)	0 (0)	0.13
	Asthma	12 (6.4)	0 (0)	12 (9.2)	0.02
**Initial vital parameters**					
RR (breath/min), mean ± SD		30 ± 10	35 ± 8	29 ± 10	<0.0001
Sp0_2_ (%), mean ± SD		85 ± 11	79 ± 10	88 ± 11	<0.0001
HR (beat/min), mean ± SD		105 ± 27	115 ± 21	100 ± 28	<0.0005
SBP (mmHg), mean ± SD		146 ± 34	154 ± 38	143 ± 32	0.09
DBP (mmHg), mean ± SD		82 ± 23	86 ± 25	80 ± 22	0.20
GCS, median (IQR)		15 (15–15)	15 (14–15)	15 (15–15)	0.69
GCS, *n* (%)					0.9
	3–8	13 (7)	4 (7.1)	9 (6.9)	
	9–12	16 (8.6)	4 (7.1)	12 (9.2)	
	13–15	158 (84)	48 (86)	110 (84)	
**Final vital parameters**^**b**^					
RR (breath/min), mean ± SD		27 ± 8	29 ± 8	26 ± 9	0.0421
SpO_2_ (%), mean ± SD		95 ± 5	96 ± 5	95 ± 5	0.0641
HR (beat/min), mean ± SD		101 ± 24	103 ± 28	100 ± 22	0.1081
SBP (mmHg), mean ± SD		135 ± 29	138 ± 30	134 ± 28	0.6171
DBP (mmHg), mean ± SD		77 ± 17	79 ± 17	76 ± 17	0.8025
GCS score, median (IQR)		15 (15–15)	15 (15–15)	15 (15–15)	0.5958
GCS, n (%)					0.85
	3–8	13 (7)	3 (5.4)	10 (7.6)	
	9–12	13 (7)	4 (7.1)	9 (6.9)	
	13–15	161 (86)	49 (88)	112 (86)	
**Treatment, n (%)**					
Oxygen		141 (75)	45 (80)	96 (73)	0.30
Intubation		5 (2.7)	1 (1.8)	4 (3.1)	0.53
Drugs					
	Salbutamol	65 (35)	20 (36)	45 (34)	0.87
	Isosorbide dinitrate	34 (18)	16 (29)	18 (24)	0.022
	Furosemide	28 (15)	14 (25)	14 (11)	0.023
	Morphine sulphate	12 (6.4)	6 (11)	6 (4.6)	0.19
	Ipratropium bromide	6 (3.2)	5 (8.9)	1 (0.8)	0.010
	Methylprednisolone	1 (0.5)	0 (0)	1 (0.8)	–
**Outcome, n (%)**					
Final diagnosis^c^					
	APE	45 (24)	20 (21)	25 (18)	0.015
	COPD exacerbation	36 (19)	12 (33)	24 (67)	0.62
	Pneumonia	59 (32)	22 (39)	37 (28)	0.14
	ARDS	0	0	0	–
	Asthma	6 (3.2)	0	6 (4.6)	0.10
48-hour mortality		13 (7)	6 (11)	7 (5.3)	0.186

^a^Overlapping presumptive diagnosis of COPD-APE for 10 patients (including 7 in the NIV group)

^b^There was a statistically significant (*p* < 0.05 for all) decrease in RR, SBP, DBP, and HR, as well as an increase in SpO_2_ in both NIV and non-NIV groups. There was no statistically significant difference for GCS

^c^Overlapping presumptive diagnosis of COPD-APE for 3 patients (including one in the NIV group)

Forty-one (31%) of the 131 included patients that were not treated with NIV still fulfilled the indication criteria for NIV. Table 2 shows their general and clinical characteristics. NIV was stopped before hospital admission in 9 (16%) of the 56 patients (Table 3). The reasons were lack of compliance or patient discomfort (*n* = 5), a technical problem (*n* = 2), persisting desaturation (*n* = 1), and vomiting (*n* = 1). After premature cessation of NIV, one patient underwent prehospital endotracheal intubation, and 1 was ventilated using a bag-mask.

**Table 2:** Characteristics of the population with a prehospital NIV indication (*n* = 97)

		Total (*n* = 97)	NIV started(*n* = 56; 58%)	NIV not started(*n* = 41; 42%)	***p*** Value
**Characteristics**					
Age (year), mean ± SD		78 ± 9	77 ± 12	81 ± 11	0.11
Male, n (%)		96 (51)	28 (29)	68 (70)	0.81
Weight (kg), mean ± SD		63 ± 29	62 ± 31	64 ± 28	0.78
NACA, n (%)					<0.0001
	3	12 (21)	0 (0)	12 (100)	
	4	34 (35)	17 (50)	17 (50)	
	5	48 (49)	38 (79)	10 (21)	
	6	2 (2)	1 (50)	3 (50)	
	7	1 (1)	0 (0)	1 (100)	
**Time interval (minutes), median (IQR)**					
Time to start		2.7 (2.0–3.6)	2.9 (2.0–4.0)	2.7 (1.9–3.3)	0.32
Time to scene		5.7 (4.0–7.9)	5.3 (3.5–7.2)	6.0 (4.8–8.1)	0.11
On-scene time		22 (16–27)	22 (18–27)	20 (14–25)	0.14
Time to hospital		9 (5–14)	8 (4–11)	13 (9–20)	<0.0001
**Presumed diagnosis, n (%)**					
	APE^a^	50 (52)	30 (54)	20 (49)	0.64
	COPD^a^ exacerbation	44 (45)	20 (36)	24 (59)	0.026
	Pneumonia	25 (26)	15 (27)	10 (24)	0.79
	ARDS	1 (1)	1 (1)	0 (0)	0.39
	Asthma	0 (0)	0 (0)	0 (0)	–
**Initial vital parameters**					
RR (breath/min), mean ± SD		35 ± 9	35 ± 8	34 ± 11	0.55
Sp0_2_ (%), mean ± SD		82 ± 11	80 ± 10	86 ± 11	0.0006
HR (beat/min), mean ± SD		108 ± 27	115 ± 21	98 ± 31	0.0023
SBP (mmHg), mean ± SD		153 ± 37	154 ± 38	152 ± 36	0.89
DBP (mmHg), mean ± SD		84 ± 24	86 ± 25	81 ± 22	0.37
GCS, median (IQR)		15 (15–15)	15 (14–15)	15 (15–15)	0.14
GCS, n (%)					0.19
	3–8	6 (6)	4 (67)	2 (33)	
	9–12	7 (7)	4 (57)	3 (43)	
	13–15	84 (86)	48 (57)	36 (43)	
**Final vital parameters**^**b**^					
RR (breath/min), mean ± SD		30 ± 8	29 ± 8	31 ± 8	0.27
SpO_2_ (%), mean ± SD		95 ± 6	96 ± 6	94 ± 7	0.29
HR (beat/min), mean ± SD		101 ± 25	103 ± 28	98 ± 19	0.39
SBP (mmHg), mean ± SD		138 ± 28	138 ± 30	139 ± 26	0.87
DBP (mmHg), mean ± SD		78 ± 18	79 ± 17	77 ± 19	0.73
GCS score, median (IQR)		15 (15–15)	15 (15–15)	15 (15–15)	0.48
GCS, n (%)					0.77
	3–8	4 (4)	3 (75)	1 (25)	
	9–12	7 (7)	4 (57)	3 (43)	
	13–15	86 (89)	49 (57)	37 (43)	
**Treatment, n (%)**					
Oxygen		80 (82)	45 (80)	35 (85)	0.52
Intubation		2 (1.0)	1 (1.8)	1 (2.4)	–
Drugs					
	Salbutamol	40 (41)	20 (36)	20 (49)	0.22
	Isosorbide dinitrate	27 (28)	16 (29)	11 (27)	1.00
	Furosemide	22 (23)	14 (25)	8 (20)	0.63
	Morphine sulfate	8 (8.2)	6 (11)	2 (4.9)	0.46
	Ipratropium bromide	5 (5.1)	5 (8.9)	0 (0)	0.07
**Outcome**					
ABG at hospital admission					
	pH	7.33 ± 0.10	7.30 ± 0.11	7.37 ± 0.07	0.0165
	PaO2 (mmHg), mean ± SD	101 ± 77	109 ± 87	92 ± 63	0.11
	PaCO2 (mmHg), mean ± SD	45 ± 17	47 ± 21	43 ± 11	0.38
	Lactate (mmol/L), mean ± SD	2.4 ± 2.0	2.8 ± 2.1	1.9 ± 1.8	0.0254
	Bicarbonates (mmol/L), mean ± SD	24 ± 5	23 ± 6	25 ± 4	0.06
Final diagnosis^c^, n (%)					
	APE	36 (37)	20 (36)	16 (39)	0.74
	COPD exacerbation	24 (25)	12 (21)	12 (29)	0.38
	Pneumonia	32 (33)	22 (39)	10 (24)	0.12
ICU admission in the first hour after hospital admission, n (%)		9 (12)	8 (22)	1 (3)	0.010
Intubation in the first hour after hospital admission, n (%)		4 (5.4)	4 (11)	0 (0)	0.035
48-hour mortality, n (%)		8 (8.3)	6 (11)	2 (4.9)	0.30

^a^Overlapping presumptive diagnosis of COPD-APE for 10 patients (including 7 in the NIV group)

^b^There was a statistically significant (*p* < 0.05 for all) decrease in RR and SBP, as well as an increase in SpO_2_ in both NIV and non-NIV groups. There was a statistically significant decrease in DBP and HR (*p* < 0.05 for both) in the NIV group but not in the non-NIV group. There was no statistically significant difference for GCS

^c^Overlapping presumptive diagnosis of COPD-APE for 3 patients (including 1 in the NIV group)

**Table 3:** Characteristics of the patients with NIV (*n* = 56)

		Total (*n* = 56)	NIV continued (*n* = 47; 84%)	NIV stopped (*n* = 9; 16%)	***p*** Value
**Characteristics**					
Age (year), mean ± SD		77 ± 12	77 ± 13	77 ± 10	0.70
Male, n (%)		28 (50)	23 (50)	5 (56)	0.06
Weight (kg), mean ± SD		61.9 ± 31	62.0 ± 31.0	61.1 ± 33.1	0.95
**NIV clinical indication, n (%)**					
	SpO_2_<90%	50 (89)	43 (91)	0.22	0.22
	RR>25 breath/min	52 (93)	44 (94)	0.61	0.61
	Use of accessory muscles^a^	45 (80)	37 (79)	0.48	0.48
**Time interval (minutes), median (IQR)**					
On-scene time		22 (18–27)	22 (18–27)	23 (18–33)	0.73
Time to hospital		7.6 (4.4–11.0)	7.3 (4.4–11.0)	9.0 (13.6–3.8)	0.67
NIV duration		21 (15–29)	21 (15–30)	11 (7–15)	0.36
**Presumed diagnosis, n (%)**					0.26
	APE	26 (46)	24 (51)	2 (7.7)	
	COPD exacerbation	17 (30)	12 (26)	5 (56)	
	Pneumonia	9 (16)	8 (17)	1 (11)	
	Other^b^	4 (7.1)	3 (6.4)	1 (11)	
**Initial vital parameters**					
RR (breath/min), mean ± SD		35 ± 8	35 ± 8	36 ± 6	0.63
Sp0_2_ (%), mean ± SD		80 ± 9	80 ± 10	78 ± 11	0.72
HR (beat/min), mean ± SD		115 ± 21	116 ± 20	110 ± 26	0.75
SBP (mmHg), mean ± SD		154 ± 38	155 ± 38	148 ± 42	0.85
DBP (mmHg), mean ± SD		8625	87 ± 25	80 ± 25	0.48
GCS, median (IQR)		15 (14–15)	15 (14–15)	15 (15–15)	0.27
GCS, n (%)					0.60
	3–8	4 (7)	4 (100)	0	
	9–12	4 (7)	3 (75)	1 (25)	
	13–15	48 (86)	47 (84)	9 (16)	
**Final vital parameters**^**d**^					
RR (breath/min), mean ± SD		29 ± 8	28 ± 7	31 ± 9	0.39
SpO_2_, mean ± SD		96 ± 5	96 ± 3	91 ± 8	0.06
HR (beat/min), mean ± SD		103 ± 28	103 ± 28	100 ± 29	0.88
SBP (mmHg), mean ± SD		138 ± 30	136 ± 28	147 ± 43	0.34
DBP (mmHg), mean ± SD		79 ± 17	78 ± 16	84 ± 25	0.75
GCS score, median (IQR)		15 (15–15)	15 (14–15)	15 (15–15)	0.45
GCS, n (%)					0.49
	3–8	3 (5)	2 (66)	1 (33)	
	9–12	4 (7)	4 (100)	0	
	13–15	49 (88)	41 (84)	8 (16)	
**Treatment, n (%)**					
Intubation		1 (1.8)	0 (0)	1 (11)	–
Drugs					
	Salbutamol	20 (36)	16 (34)	4 (44)	0.70
	Isosorbide dinitrate	16 (29)	14 (30)	2 (13)	1.00
	Furosemide	14 (25)	14 (30)	0 (0)	0.09
	Morphine sulfate	10 (18)	5 (11)	1 (17)	1.00
	Ipratropium bromide	9 (16)	4 (8.5)	1 (20)	1.00
	Methylprednisolone	0 (0)	0 (0)	0 (0)	–
**NIV initial setting**					
	PEEP (mmHg), mean ± SD	5.1 ± 1.5	5.2 ± 1.7	5 ± 0	0.29
	Pressure support (mmHg), mean ± SD	6.1 ± 2.6	6.3 ± 2.6	5.1 ± 2.3	0.24
	FiO_2_ (%), mean ± SD	92 ± 20	91 ± 21	100 ± 0	0.16
**NIV setting at hospital admission**					
	FiO_2_ (%), mean ± SD	–	73 ± 27	–	–
	PEEP (mmHg), mean ± SD	–	5.8 ± 2.7	–	–
	Inspiratory pressure (mmHg), mean ± SD	–	7.9 ± 3.8	–	–
**D-VOS**^**e**^					
	Initial	8.6 ± 1.6	8.4 ± 1.7	9.2 ± 1.2	0.27
	At hospital admission or NIV cessation	5.0 ± 2.1	4.4 ± 1.8	7.3 ± 1.6	0.0037
**48-h follow-up**					
48-hour mortality, n (%)		6 (11)	4 (8.5)	2 (22)	0.22
**Final diagnosis,**^**c**^ **n (%)**					
	Acute pulmonary edema	20 (36)	18 (38)	2 (22)	0.36
	COPD	12 (21)	10 (21)	2 (22)	0.95
	Pneumonia	22 (39)	20 (43)	2 (22)	0.25

^a^The use of accessory muscles was never the only clinical indication to NIV, as it was always associated with either hypoxemia or tachypnea

^b^Bronchoaspiration (n = 2); pulmonary embolism (n = 1); hyperviscosity in acute myeloid leukemia (n = 1)

^c^Overlapping presumptive diagnosis of COPD-APE for 3 patients (including one in the NIV stopped group)

^d^n = 34.

^e^*p* < 0.0001 for comparison between D-VOS on site and at hospital and 0.0523 between initial and when stopped. A D-VOS was available on site and at hospital for 25 patients, and on site and at NIV cessation for 6 patients

**Fig. 2:**
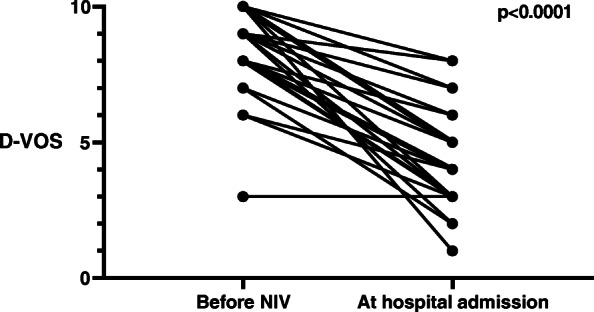
Dyspnea verbal ordinal scale (D-VOS) before noninvasive ventilation (NIV) and at hospital admission for patients with NIV

The mean D-VOS score at hospital in the NIV group was significantly lower than the initial D-VOS score (4.4 ± 1.8 vs 8.4 ± 1.7; *p* < 0.0001, Figure 2). Most patients (*n* = 44; 94%) continued NIV in hospital.

## Discussion

In our PEMS, 30% of patients with acute respiratory distress, according to study criteria, were treated with NIV from January 1, 2019, to December 31, 2019. 31% of patients managed without NIV had an indication for it, and the NIV failure rate was 16%. To our knowledge, the present study is the first to comprehensively assess the process of initiating NIV in the prehospital setting, including the description of patients for whom NIV was not initiated and the reasons why.

The most frequently reported presumed causes of respiratory distress in our case load were APE, COPD exacerbation, and pneumonia, which is consistent with the findings of previous studies [7]. In the NIV group, the proportion of presumed diagnosis of APE and COPD exacerbation was higher than in the non-NIV group, which is consistent with the proposed indication criteria for prehospital NIV in our setting. Interestingly, the third main presumed diagnosis was pneumonia in 9 (16%) patients in the NIV group. As the role of NIV in the management of respiratory failure caused by pneumonia only is debated, we did not include pneumonia in our internal recommendations as an indication for NIV. It seems, however, that patients with pneumonia could benefit from NIV if they have cardiac or respiratory comorbidities, and NIV may be proposed in select situations, which may have been the case for those patients [17–19]. A diagnosis of pneumonia was even more frequent when considering 48-hour diagnosis (22 patients, 39%) in the NIV group, whereas the number of final diagnoses of APE and COPD exacerbations was lower than initially suspected. Making an accurate etiological diagnosis of dyspnea in the prehospital setting is difficult because of the absence of pathognomonic signs or symptoms [20]. If the field assessment of dyspnea from a cardiac or non-cardiac cause has some concordance with the emergency department diagnosis, a more precise diagnosis may be more problematic, notably in the case of multiple etiologies [3]. The accuracy between prehospital respiratory distress diagnoses by paramedics or prehospital physicians and emergency physicians is moderate [21–26]. To our knowledge, there is no study comparing the accuracy of diagnoses of respiratory distress between paramedics and prehospital physicians. In our setting, prehospital physicians, by virtue of their experience and training, have an advanced clinical judgment. The etiology of the respiratory distress may be better defined, allowing for more precise decision on whether NIV would be indicated or not, but this remains to be demonstrated. Given the difficulty of establishing a precise diagnosis, prehospital NIV may be more frequently started on the basis of symptoms than on the actual cause of the dyspnea.

Among the patients who did not receveived prehospital NIV, 31% nevertheless fulfilled the predefined indication criteria. Compared with patients who received NIV, patients with an indication for NIV who did not receive NIV had a higher initial SpO_2_ (86 vs 80%), a lower HR (98 vs 115 beats/min), and a tendency to be less clinically severe according to the NACA score. The severity of the case, especially the SpO_2_ level, seemed to be the element that determined the NIV indication, the most hypoxemic patients being more likely to be treated with NIV.

Although a short distance to the hospital should not prevent the use of NIV, it is known that the mortality from respiratory causes is increased as the distance to the hospital increases [14, 27]. We could therefore have expected that physicians would decide not to start NIV because of a short transport time to hospital. Nonetheless, the transport time to hospital did not seem to influence the NIV decision in our study. The transport time to hospital was indeed even higher for patients without NIV than it was for those in the NIV group (13 vs 8 min). This may be explained by several unmeasured variables, including the potentially more frequent use of lights and siren to transport the more severe cases from the NIV group, although priority signals do not seem to be a big time-saver [1]. A prospective study would be necessary to analyze more precisely whether other factors, relative to logistical issues or to the physicians themselves, could also partially explain some part of this “undertreatment”.

The potential advantages of NIV in the management of APE and COPD exacerbation include improvement of vital signs, lowering of the intubation and ICU admission rates, diminution of the hospital length of stay as well as health care costs [8, 10, 11, 28, 29]. Most studies have reported such findings in the hospital setting. Although the relatively low number of patients in the present study is not sufficient to draw reliable conclusions about strong outcomes such as mortality, some of our results may nonetheless support the benefit of NIV treatment. A first argument is the evolution of the vital signs. As the initial vital signs (SpO_2_, RR, HR) deviated more from normal in the NIV group than they were in the study population in general or those with an indication for NIV, the NIV group underwent significant improvement at hospital arrival, suggesting that patients who received NIV benefited from it. This may be especially relevant, as failure to normalize vital signs has been correlated with poor outcome [30]. The second argument in favor of prehospital NIV is the subjective improvement of dyspnea in the NIV group. In the group of patients treated with NIV, the final D-VOS score was lower than the initial one. This improvement was even higher in patients for whom NIV was continued until hospital admission (hospital D-VOS score of 4.4 vs initial D-VOS score of 8.4) than it was in the group for whom NIV was interrupted. These results are not only statistically but also clinically significant, with an absolute reduction in the D-VOS score of 4 in patients for whom NIV was continued to hospital admission. This is important, as the level of dyspnea correlates to clinical parameters used in emergency departments [31]. Furthermore, studies of the multidimensional extent of dyspnea have revealed an important affective aspect, and laboratory experience has shown, for example, that respiratory work during effort is less unpleasant than air hunger [32, 33]. Finally, the ABG profile at hospital admission in the NIV group was usually favorable, when considering the mild respiratory acidosis without major hypoxemia.

The potential benefit of NIV has to be weighed against the potential harm. There was no significant difference in 48-hour mortality between the groups, including in patients for whom NIV had to be stopped. The failure rate of NIV was 16% in the present study, which is less than the 26% observed in the prehospital field and the 17% to 30% in the ICU [34, 35]. In 2003, a study showed a similar failure rate (17%), but it included only COPD exacerbation treated with continuous positive airway pressure [36]. Failures are explained more by management of the ventilator, including patient discomfort and technical problems, and less by deterioration of the clinical condition, such as persistent desaturation and vomiting, which are expected complications [28, 37]. We did not detect any hemodynamic instability leading to cessation of NIV, and the only patient with persistent hypoxemia was successfully intubated. Another potential risk for some patients may be the prolongation of the on-scene time. However, in our study, the on-scene time was similar in the NIV group as it was in the other groups, suggesting that NIV did not affect on-scene time, as previously described [11, 38].

Our internal recommendations concerning the NIV in prehospital settings were continuously reassessed during the COVID pandemic, taking into account scientific knowledge and local epidemiology. Initially stopped at the beginning of the pandemic because NIV was regarded as a potential risk of contamination by aerosolization, we have now resumed its use for the initial indications (APE and COPD exacerbations) with or without a suspicion of SARS-CoV-2 infection. The use of full personal protective equipment is systematic when using NIV.

A main strength of the present study is that we comprehensively assessed the process for initiating NIV in the prehospital setting, including in patients for whom NIV was not initiated, and provided a hospital 48-hour outcome and diagnosis. Another strength is the precise description of the population treated with NIV, including an evaluation of dyspnea intensity with the D-VOS, as well as the hospital ABG analysis.

Our study also has several limitations, most of them pertaining to its retrospective condition. First, the improvement in vital parameters and D-VOS score in the NIV group may have been the consequence of co-treatments. Regarding adjuvant treatment, recommendations support the administration of oxygen and medication, namely, treatment of the precipitating cause and diuretics for APE and a short-acting inhaled beta2-agonist for COPD exacerbation [5, 6]. Nonetheless, we did not find any significant difference in the drugs administered to the NIV group compared with those administered to patients without NIV who had an indication for NIV, or among those administered to patients with NIV depending on whether NIV had to be stopped before hospital admission or not. Another limitation is the lack of important clinical information (D-VOS score) and outcomes (1-hour intubation or admission to ICU, ABG analysis), for patients for whom NIV had not been considered. A 48-hour outcome was, however, available for almost every patient, which also included the 48-hour diagnosis, which is rarely available in prehospital studies [7, 9]. By definition, the NACA score of patients who received NIV is 5; this made patients from the NIV group appear to be more severe. Clinical variables did, however, give a good indication of severity. Distance to hospital is in general very short in Lausanne’s PEMS district. Therefore clinical improvement with NIV and worsening without NIV could be underestimated and the results may not be transposed in larger areas. Another limitation of our study is the large number of group comparisons performed, which could lead to some important inflation of the type I error (and thus of the risk of getting “false significant” results). The most radical (and often overly conservative) way to adjust for this would be to perform a Bonferroni correction, where one can simply multiply a *p*-value by the number of tests performed (in our case ca. 100) before comparing it with 0.05. In our situation, this means that original *p*-values below 0.005 could still be considered statistically significant. Finally, it was not possible to retrospectively reliably assess the use of accessory muscles as a criterion for NIV indication, as it is not specifically recorded in the prehospital chart. However, this criterion was seldom reported alone in the NIV group but rather in conjunction with the other parameters (desaturation and tachypnea) that indicate NIV.

## Conclusion

In our study, patients with acute respiratory distress treated with NIV tended to be more clinically severe. These patients improved more quickly than did those with an indication for NIV who did not receive it. The patient who had to stop NIV prematurely felt more dyspneic. The major causes of NIV interruption were problems with the management of the ventilator and patient discomfort. A prospective study could contribute to the understanding of why patients with a suspected diagnosis of APE and COPD are not treated with NIV.

## Data Availability

The datasets generated and/or analysed during the current study are not publicly available due to the absence of specific ethical committee approval regarding this specific point. The datasets may be available from the corresponding author on reasonable request and providing specific ethical committee approval is obtained.

## Abbreviations

ABG: arterial blood gas
APE: acute pulmonary edema
ARDS: acute respiratory distress syndrome
COPD: chronic obstructive pulmonary disease
DBP: diastolic blood pressures
BiPAP: bi-level positive airway pressure
D-VOS: dyspnea verbal ordinal scale
FiO_2_: fraction of inspired oxygen
GCS: Glasgow Coma Scale
HCO_3_: bicarbonates
HR: heart rate
ICU: intensive care unit
IQR: interquartile range
NACA: National Advisory Committee for Aeronautics
NIV: noninvasive ventilation
PaCO_2_: arterial partial pressure of carbon dioxide
PaO2: arterial partial pressure of oxygen
PEEP: positive end expiratory pressure
PEMS: physician-based emergency medical services
PS: pressure support
RR: respiratory rate
SBP: systolic blood pressures
SD: standard deviation
SpO_2_: oxygen saturation

## References

[1] Dami F, Pasquier M, Carron PN (2014). Use of lights and siren: is there room for improvement?. Eur J Emerg Med..

[2] Prekker ME, Feemster LC, Hough CL, Carlbom D, Crothers K, Au DH (2014). The epidemiology and outcome of prehospital respiratory distress. Acad Emerg Med..

[3] Pozner CN, Levine M, Shapiro N, Hanrahan JP (2003). Concordance of field and emergency department assessment in the prehospital management of patients with dyspnea. Prehosp Emerg Care..

[4] Rochwerg B, Brochard L, Elliott MW, Hess D, Hill NS, Nava S (2017). Official ERS/ATS clinical practice guidelines: noninvasive ventilation for acute respiratory failure. Eur Respir J..

[5] Ponikowski P, Voors AA, Anker SD, Bueno H, Cleland JG, Coats AJ (2016). 2016 ESC Guidelines for the diagnosis and treatment of acute and chronic heart failure: The task force for the diagnosis and treatment of acute and chronic heart failure of the European Society of Cardiology (ESC). Eur J Heart Fail..

[6] Vogelmeier CF, Criner GJ, Martinez FJ, Anzueto A, Barnes PJ, Bourbeau J (2017). Global strategy for the diagnosis, management, and prevention of chronic obstructive lung disease 2017 report. Am J Respir Crit Care Med..

[7] Roessler MS, Schmid DS, Michels P, Schmid O, Jung K, Stöber J (2012). Early out-of-hospital non-invasive ventilation is superior to standard medical treatment in patients with acute respiratory failure: a pilot study. Emerg Med J..

[8] Park M, Sangean MC, Volpe Mde S, Feltrim MI, Nozawa E, Leite PF (2004). Randomized, prospective trial of oxygen, continuous positive airway pressure, and bilevel positive airway pressure by face mask in acute cardiogenic pulmonary edema. Crit Care Med..

[9] Schmidbauer W, Ahlers O, Spies C, Dreyer A, Mager G, Kerner T (2011). Early prehospital use of non-invasive ventilation improves acute respiratory failure in acute exacerbation of chronic obstructive pulmonary disease. Emerg Med J..

[10] Williams TA, Finn J, Perkins GD, Jacobs IG (2013). Prehospital continuous positive airway pressure for acute respiratory failure: a systematic review and meta-analysis. Prehosp Emerg Care..

[11] Gartner BA, Fehlmann C, Suppan L, Niquille M, Rutschmann OT, Sarasin F (2020). Effect of noninvasive ventilation on intubation risk in prehospital patients with acute cardiogenic pulmonary edema: a retrospective study. Eur J Emerg Med..

[12] Rapport d’activités—Association des communes de la région lausannoise. 2019.

[13] Boussen S, Gainnier M, Michelet P (2013). Evaluation of ventilators used during transport of critically ill patients: a bench study. Respir Care..

[14] Hensel M, Strunden MS, Tank S, Gagelmann N, Wirtz S, Kerner T (2019). Prehospital non-invasive ventilation in acute respiratory failure is justified even if the distance to hospital is short. Am J Emerg Med..

[15] Darioli V, Taffé P, Carron PN, Dami F, Valloton L, Yersin B (2019). Evaluation of the discriminative performance of the prehospital national advisory committee for aeronautics score regarding 48h mortality. Eur J Emerg Med..

[16] Lansing RW, Moosavi SH, Banzett RB (2003). Measurement of dyspnea: word labeled visual analog scale vs. verbal ordinal scale. Respir Physiol Neurobiol..

[17] Carrillo A, Gonzalez-Diaz G, Ferrer M, Martinez-Quintana ME, Lopez-Martinez A, Llamas N (2012). Non-invasive ventilation in community-acquired pneumonia and severe acute respiratory failure. Intensive Care Med..

[18] Nicolini A, Piroddi IM, Barlascini C, Senarega R (2014). Predictors of non-invasive ventilation failure in severe respiratory failure due to community acquired pneumonia. Tanaffos..

[19] British Thoracic Society Standards of Care Committee (2002). Non-invasive ventilation in acute respiratory failure. Thorax..

[20] Renier W, Winckelmann KH, Verbakel JY, Aertgeerts B, Buntinx F (2018). Signs and symptoms in adult patients with acute dyspnea: a systematic review and meta-analysis. Eur J Emerg Med..

[21] Koivulahti O, Tommila M, Haavisto E (2020). The accuracy of preliminary diagnoses made by paramedics - a cross-sectional comparative study. Scand J Trauma Resusc Emerg Med..

[22] Christie A, Costa-Scorse B, Nicholls M, Jones P, Howie G (2016). Accuracy of working diagnosis by paramedics for patients presenting with dyspnoea. Emerg Med Australas..

[23] Cummins NM, Dixon M, Garavan C, Landymore E, Mulligan N, O’Donnell C (2013). Can advanced paramedics in the field diagnose patients and predict hospital admission ?. Emerg Med J..

[24] Williams TA, Finn J, Celenza A, Jacobs IG (2013). Paramedic identification of acute pulmonary edema in a metropolitan ambulance service. Prehosp Emerg Care..

[25] Schewe JC, Kappler J, Dovermann K, Graeff I, Ehrentraut SF, Heister U (2019). Diagnostic accuracy of physician-staffed emergency medical teams: a retrospective observational cohort study of prehospital versus hospital diagnosis in a 10-year interval. Scand J Trauma Resusc Emerg Med..

[26] Ramadanov N, Klein R, Laue F, Behringer W. Diagnostic agreement between prehospital emergency and in-hospital physicians. Emerg Med Int. 2019; 10.1155/2019/3769826. 10.1155/2019/3769826 PMC6507260 31179130

[27] Nicholl J, West J, Goodacre S, Turner J (2007). The relationship between distance to hospital and patient mortality in emergencies: an observational study. Emerg Med J..

[28] Mal S, McLeod S, Iansavichene A, Dukelow A, Lewell M (2014). Effect of Out-of-hospital noninvasive positive-pressure support ventilation in adult patients with severe respiratory distress: a systematic review and meta-analysis. Ann Emerg Med..

[29] Thokala P, Goodacre S, Ward M, Penn-Ashman J, Perkins GD (2015). Cost-effectiveness of out-of-hospital continuous positive airway pressure for acute respiratory failure. Ann Emerg Med..

[30] Levin N, Horton D, Sanford M, Horne B, Saseendran M, Graves K (2020). Failure of vital sign normalization is more strongly associated than single measures with mortality and outcomes. Am J Emerg Med..

[31] Kendrick KR, Baxi SC, Smith RM (2000). Usefulness of the modified 0-10 Borg scale in assessing the degree of dyspnea in patients with COPD and asthma. J Emerg Nurs..

[32] Banzett RB, Pedersen SH, Schwartzstein RM, Lansing RW (2008). The affective dimension of laboratory dyspnea air hunger is more unpleasant than work/effort. Am J Respir Crit Care Med..

[33] Banzett RB, O’Donnell CR, Guilfoyle TE, Parshall MB, Schwartzstein RM, Meek PM (2015). Multidimensional dyspnea profile: an instrument for clinical and laboratory research. Eur Respir J..

[34] Bruge P, Jabre P, Dru M, Jbeili C, Lecarpentier E, Khalid M (2008). An observational study of noninvasive positive pressure ventilation in an out-of-hospital setting. Am J Emerg Med..

[35] Antonelli M, Conti G, Moro ML, Esquinas A, Gonzalez-Diaz G, Confalonieri M (2001). Predictors of failure of noninvasive positive pressure ventilation in patients with acute hypoxemic respiratory failure: a multi-center study. Intensive Care Med..

[36] Carlucci A, Delmastro M, Rubini F, Fracchia C, Nava S (2003). Changes in the practice of non-invasive ventilation in treating COPD patients over 8 years. Intensive Care Med..

[37] Pandor A, Thokala P, Goodacre S, Poku E, Stevens JW, Ren S (2015). Pre-hospital non-invasive ventilation for acute respiratory failure: a systematic review and cost-effectiveness evaluation. Health Technol Assess..

[38] Bakke SA, Botker MT, Riddervold IS, Kirkegaard H, Christensen EF (2014). Continuous positive airway pressure and noninvasive ventilation in prehospital treatment of patients with acute respiratory failure: a systematic review of controlled studies. SJTREM..

